# Adrenal Incidentalomas: Should We Operate on Small Tumors in the Era of Laparoscopy?

**DOI:** 10.1155/2014/658483

**Published:** 2014-04-10

**Authors:** Michał Pędziwiatr, Michał Natkaniec, Mikhail Kisialeuski, Piotr Major, Maciej Matłok, Damian Kołodziej, Anna Zub-Pokrowiecka, Piotr Budzyński, Andrzej Budzyński

**Affiliations:** Department of Endoscopic, Metabolic and Soft Tissue Tumors Surgery, 2nd Department of General Surgery, Jagiellonian University, Kopernika 21, 31-501 Kraków, Poland

## Abstract

Tumor size smaller than 4 cm as an indication for surgical treatment of incidentaloma is still a subject of discussion. Our aim was the estimation of the incidence of malignancy and analysis of treatment outcomes in patients with incidentaloma smaller than 4 cm in comparison to bigger lesions. 132 patients who underwent laparoscopic adrenalectomy for nonsecreting tumors were divided into two groups: group 1 (55 pts., size < = 40 mm) and group 2 (77 pts., size > 40 mm). Operation parameters and histopathological results were analyzed. No differences in group characteristics, mean operation time, and estimated blood loss were noted. Complications in groups 1 and 2 occurred in 3.6% and 5.2% of patients, respectively (*P* = 0.67). Malignancy in groups 1 and 2 was present in 1 and 6 patients, respectively (*P* = 0.13). Potentially malignant lesions were identified in 4 patients in group 1 and 4 patients in group 2 (*P* = 0.39). The results do not allow for straightforward recommendations for surgical treatment of smaller adrenal tumors. The safety of laparoscopy and minimal, but impossible to omit, risk of malignancy support decisions for surgery. On the other hand, the risk of malignancy in smaller adrenal tumors is lower than surgical complications, which provides an important argument against surgery.

## 1. Introduction

Incidentalomas are a group of hormonally inactive adrenal tumors incidentally found during imaging studies performed in patients due to symptoms unrelated to adrenal tumors. Patients diagnosed and treated for neoplastic disorders of other organs are excluded as metastasis to the adrenal glands should be suspected at first place in that group of patients [1]. The prevalence of incidentaloma grows with the patients' age [2]. A steady increase in this diagnosis can also be observed over the last few decades. According to the latest data such lesions can be found in 4–10% of patients [1, 3, 4]. This greater incidence of hormonally inactive tumors results at least partially from the higher quality and precision of imaging techniques available today compared to those at the end of the previous century. Nowadays imaging is based on high resolution computed tomography (CT) or magnetic resonance imaging (MRI) as opposed to ultrasound (US), which was used for diagnosis in the past. However, it also seems impossible to exclude the fact that there is a real increase in the incidence of incidentaloma in the general population [1, 5].

Even though current guidelines clearly list the indications for adrenalectomy (tumor size, abrupt growth found in follow-up studies, or radiologic appearance suggesting a malignant lesion), the final decision whether to operate or to follow up belongs to the surgeon [6]. The greatest concern is the potentially malignant character of an adrenal tumor. Adrenal cortical carcinoma, which is quite rare, is, like other even more rare malignancies, found mainly in large tumors [3]. Despite this fact, evidence of possible malignancy in smaller adrenal tumors also exists [2]. The poor prognosis associated with this type of tumors can prompt a more radical approach also in the case of smaller lesions [7–9]. Different values of tumor size as indication for surgical treatment can be found in various sources in the literature. The size quoted usually ranges from 3 to 5 cm, and one of the most commonly used is 4 cm [6, 10, 11]. With an increase in the upper range of tumor size the risk of missed potentially resectable malignancy increases. On the other hand, an overconfident belief in the safety of minimally invasive procedures may lead to an unjustified broadening of the inclusion criteria for surgery in the case of smaller tumors. This can expose a larger number of patients to postoperative complications without obvious benefits.

## 2. Aim

The aim of this study is the evaluation of the incidence of malignant or potentially malignant lesions in patients with hormonally inactive adrenal tumors smaller than 4 cm in diameter in comparison to the group of patients with lesions greater than 4 cm in diameter. The second aim was the analysis of early treatment results in relation to the tumor size.

## 3. Materials and Methods

The study focused on the retrospective analysis of patients who underwent laparoscopic lateral transperitoneal adrenalectomy between 2003 and 2013. The inclusion criterion was the presence of an adrenal tumor incidentally discovered in imaging studies (computed tomography, CT; magnetic resonance imaging, MRI; ultrasound, US). During diagnostic workout hormonal activity and active malignant processes of other origins were excluded. Patients with a previous history of malignancies were excluded as well. The main indication for adrenalectomy in case of hormonally inactive tumors was size above 40 mm. In case of smaller lesions the decision on surgery was based on significant growth in follow-up CT/MRI or a so-called malignant phenotype discovered in imaging.

In the analyzed period 468 adrenalectomies were performed at our center. In 319 patients the indication for the surgical treatment was the presence of a hormonally active adrenal tumor, in case of 17 patients, metastasis from another source. The study group consisted of the 132 remaining patients with hormonally inactive and incidentally discovered tumors. Patients were divided into two groups, according to tumor size. Group 1 included 55 patients (39 females and 16 males) with a tumor not larger than 40 mm in diameter. Group 2 included 77 patients (52 females and 25 males) with a tumor larger than 40 mm in diameter.  Table 1 presents the characteristics of the study groups. Operation time, conversion rate, perioperative complications, and histopathological results were analyzed. Blood loss was measured by volume in the suction container. This method was sufficiently accurate, as irrigation was never used in case of laparoscopic adrenalectomy. During the evaluation of the pathological results, the lesions were classified into one of 3 categories: benign, potentially malignant, and malignant. Tumors were classified as potentially malignant if they did not present overt features of malignancy intraoperatively or histologically; however, as we have known from the literature, they occasionally show malignant behavior [12].

Chi-square, Student's *t*-, and Pearson correlation tests were used in statistical analysis. *P* < 0.05 was considered significant.

## 4. Results

The characteristics of the study groups are presented in Table 1. There were no statistically significant differences between groups in regard to the gender, age, or the side where the tumor was located.

**Figure 1:**
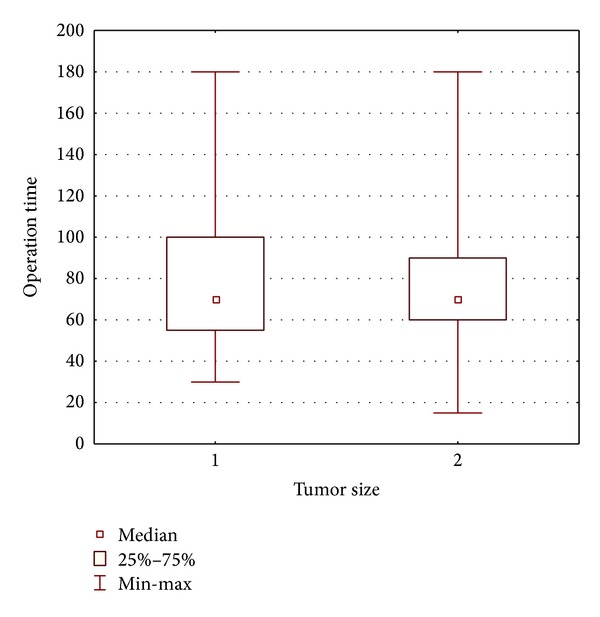
Mean operation time in studied groups.

There were no conversions to open surgery in group 1 and in group 2 conversions were necessary in two cases (*P* = 0.34). Reasons for conversions included adhesions after previous surgeries in one patient and tumor capsule rupture in a patient with an 8 cm tumor highly suspected to be malignant. Mean operation time in group 1 was 77 min., while in group 2 81 min. (*P* = 0.713; Figure 1).

**Figure 2:**
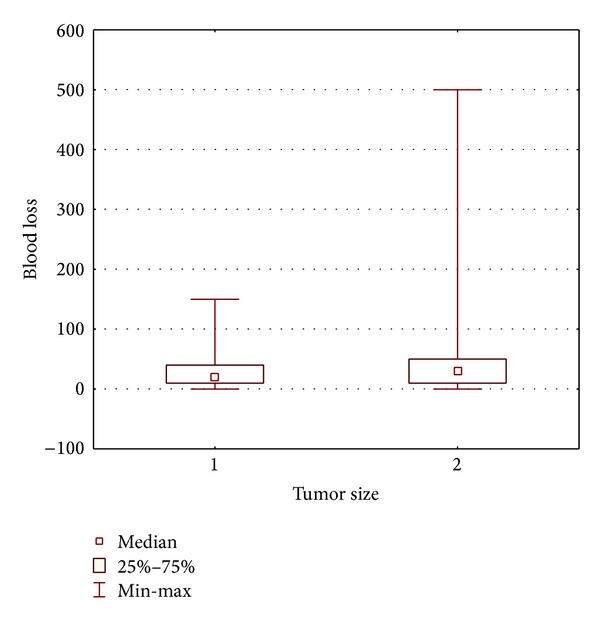
Mean estimated blood loss in studied groups.

Mean estimated blood loss in group 1 was 37 mL and 81 mL in group 2. No statistically significant difference was shown between the groups (*P* = 0.136; Figure 2).

Complications occurred in two (3.6%) patients in group 1 (wound infection and pleural effusion, respectively; 1st and 2nd grade in the Clavien-Dindo classification). Complications in group 2 occurred in four (5.2%) patients. They included diaphragm injury, inferior vena cava injury that was sutured laparoscopically during the primary procedure (3rd grade in the Clavien-Dindo classification), hematoma in the removed adrenal tumor's bed (2nd grade), and wound infection (1st grade in the Clavien-Dindo classification). The difference between the number of complications in the study groups is not statistically significant (*P* = 0.67). None of the patients required reoperation. There were no deaths in the 30-day postoperative period.

During the histopathological analysis of the results in group 1 only one malignant lesion was found (1.8%) and in four patients (7.2%) lesions that were regarded potentially malignant were found. Group 2 had six cases (7.8%) of malignant lesions and in an additional four patients tumors that were considered potentially malignant were found.  Table 2 presents the histological types of the removed adrenal tumors.

## 5. Discussion

Until the mid-1990s adrenalectomies were performed in highly specialized endocrinological surgery hospitals. The procedure was considered difficult and risky, with high morbidity and mortality rates. In 1992 Gagner was the first to report laparoscopic adrenalectomy and this significantly influenced the development of endocrine surgery [13, 14]. Even though the introduction of minimally invasive techniques theoretically should not influence the indications for the surgery, an appreciable increase in the number of adrenalectomies in many centers can be observed. A low risk of complications, shorter hospital stays, and more rapid recovery contributed to high popularity of laparoscopic adrenalectomy. Endocrinologists refer their patients more willingly to centers where laparoscopic surgery is successfully performed [15, 16]. There is now a general assumption that minimally invasive techniques provide a relatively safe and predictable way to treat adrenal tumors. There is, however, a risk that this situation can prompt surgeons to operate on patients that potentially could instead be safely followed up.

One of the greatest concerns here is the borderline tumor size that constitutes an indication for surgery [17, 18]. The upper limit of the size of tumors that can be removed by minimally invasive technique has not been clearly established. However, this problem is less controversial as there are many reports on successful laparoscopic adrenalectomy for large tumors [19–21]. The question concerning the lower limit of tumor size that constitutes an indication for the operative treatment seems to be more important. This criterion has changed several times, ranging from 2 to 6 cm. The majority of authors advocate surgery for tumors between 3 and 5 cm in diameter. It seems that most surgeons, though certainly not all of them, accepted a lower limit of the tumor size of 4 cm as an indication for the surgical treatment [3, 11].

The greatest concern in the case of hormonally inactive adrenal tumors is the potential for malignancy, which can occur even in relatively small tumors. It is well known that the incidence of cancer is associated with the size of the tumor. In lesions over 6 cm it can vary between 5% and 25% [7]. Yet, it is strikingly lower in the case of tumors smaller than 4 cm (0.8–2%) [13]. In our group, adrenal cortex cancer was found in one patient (1.8%) in group 1 and in five patients (6,5%) in group 2. These data are relevant to the reports from the literature and confirm the hypothesis that adrenal malignancy in case of small incidentalomas is rare, but still possible. However, the concern about missed malignancy may lead to a potential risk of overtreatment, as the objective incidence of these lesions is relatively low [3].

The extended indications for surgery in the case of small tumors should include their characteristics in commonly used imaging techniques. The sensitivity and specificity of CT in case of typical adrenal adenoma are 71% and 98%, respectively [22–24]. Unfortunately, even up to 30% of adenomas do not present a typical image in CT and cannot be differentiated from malignant lesions [8]. Similarly to CT, MRI in up to 10–30% of cases cannot distinguish the character of the adrenal tumor [8, 25]. The inability to reliably assess up to 1/3 of all incidentalomas presenting the so-called radiologically malignant phenotype is one of the most common factors influencing the decision about surgery. Other imaging techniques, like scintigraphy or scintigraphy with metaiodobenzylguanidine, are used rather in the case of hormonally active tumors, while PET CT may be of some value in the case of suspected metastases in patients with a history of malignancy [1]. These methods, however, are not routinely used in the diagnostics of incidentalomas and have little influence on the decision about surgery.

The outcome of the follow-up in patients with lesions that were not operated on is another important issue. The review of 21 studies by Kapoor et al. included 1690 patients with incidentalomas smaller than 4 cm treated conservatively. It was noticed that, in follow-up (1.5 to 7 years), progression in size was present in 12.5% of patients, and a decrease in tumor size was observed in 4.3% of cases. Additionally, hormonal activity appeared in 1.2% of patients. The overall risk of growth of the adrenal tumors at surveillance in a 5-year period was estimated to be 18–29% [1]. The change in the size or character of the tumor is a significant criterion indicating the need for surgery. It can be assumed that every third patient with a tumor smaller than 3 cm will eventually require surgical treatment in the future. Unfortunately, it is impossible to predict in whom changes in tumor size or character will occur.

Another factor favoring the removal of adrenal tumors smaller than 4 cm is the safety profile provided by minimally invasive techniques. It is represented by a low complication rate, short hospital stay, and an absence of adverse long-term outcomes. In our group of patients the complication rate was 3.6% and 5.1% in groups 1 and 2, respectively. There were no complications higher than grade 3 in the Clavien-Dindo classification. Conversions, similarly to complications, were not related to the size of the adrenal tumor. Additionally, the operation time and mean estimated blood loss were similar in both groups. None of the patients required reoperation. Therefore, we can assume that tumor size within the range discussed in this paper does not affect the results of surgical treatment. We may thus conclude that laparoscopic adrenalectomy is safe regardless of tumor size, and during the qualification process one should not be guided by tumor size. All these factors may lead to an increase in the overall number of adrenalectomies for reasons poorly supported by scientific evidence.

## 6. Conclusions

This study does not provide unequivocal conclusions regarding the indications for and safety of laparoscopic adrenalectomy in tumors smaller than 4 cm. The safety of laparoscopic surgery and the minimal, though impossible to omit, risk of development of malignancy provide an argument for surgical treatment. On the other hand, the fact that the risk of malignancy in adrenal tumors smaller than 4 cm is lower (1.8%) than the risk of complications related to laparoscopic adrenalectomy (3.6%) provides an important counterargument against surgery.

## Figures and Tables

**Table 1:** Characteristics of the study groups of patients.

	Group 1 (tumor < 4 cm), *n* = 55	Group 2 (tumor ≥ 4 cm), *n* = 77	*P* value
Female/male	39 (71%)/16 (29%)	52 (68%)/25 (32%)	0.69
Left side/right side	29 (53%)/26 (47%)	40 (52%)/37 (48%)	0.93
Mean age	57 years (±11.4) (24–76)	52.9 years (±14.1) (19–81)	0.15
Average tumor size	3.13 cm (±0.57) (1.2–3.8)	5.63 cm (±2.2) (4–16)	<0.001

**Table 2:** Histological types of removed lesions.

Histological type		Group 1		Group 2		*P* value
Benign	Cortical adenoma	**50 (91%)**	42 (76.4%)	**68 (88.3%)**	43 (55.8%)	0.72
	Adrenal cyst		3 (5.4%)		12 (15.6%)	
	Angiomyolipoma		2 (3.6%)		9 (11.7%)	
	Cavernous haemangioma		1 (1.8%)		0 (0%)	
	Schwannoma		1 (1.8%)		2 (2.6%)	
	Ganglioneuroma		1 (1.8%)		2 (2.6%)	

Potentially malignant	Pheochromocytoma	**4 (7.2%)**	2 (3.6%)	**3 (3.9%)**	3 (3.9%)	0.39
	Oncocytic adrenal adenoma		2 (3.6%)		0 (0%)	

Malignant	Adrenal cortex cancer	**1 (1.8%)**	1 (1.8%)	**6 (7.8%)**	5 (6.5%)	0.13
	Primary neuroectodermal tumor		0 (0%)		1 (1.3%)	

## References

[1] Kapoor A, Morris T, Rebello R (2011). Guidelines for the management of the incidentally discovered adrenal mass. *Journal of the Canadian Urological Association*.

[2] Barzon L, Sonino N, Fallo F, Palù G, Boscaro M (2003). Prevalence and natural history of adrenal incidentalomas. *European Journal of Endocrinology*.

[3] Young WF (2007). The incidentally discovered adrenal mass. *The New England Journal of Medicine*.

[4] Chavez-Rodriguez J, Pasieka JL, Sinanan M (2005). Adrenal lesions assessed in the era of laparoscopic adrenalectomy: a modern day series. *The American Journal of Surgery*.

[5] Gopan T, Remer E, Hamrahian AH (2006). Evaluating and managing adrenal incidentalomas. *Cleveland Clinic Journal of Medicine*.

[6] Burpee SE, Jossart GH, Gagner M, Holzheimer RG, Mannick JA (2001). Laparoscopic adrenalectomy. *Surgical Treatment: Evidence-Based and Problem-Oriented*.

[7] Mansmann G, Lau J, Balk E, Rothberg M, Miyachi Y, Bornstein SR (2004). The clinically inapparent adrenal mass: update in diagnosis and management. *Endocrine Reviews*.

[8] Szolar DH, Korobkin M, Reittner P (2005). Adrenocortical carcinomas and adrenal pheochromocytomas: Mass and enhancement loss evaluation at delayed contrast-enhanced CT. *Radiology*.

[9] Barnett CC, Varma DG, El-Naggar AK (2000). Limitations of size as a criterion in the evaluation of adrenal tumors. *Surgery*.

[10] Bin X, Qing Y, Linhui W, Li G, Yinghao S (2011). Adrenal incidentalomas: experience from a retrospective study in a Chinese population. *Urologic Oncology*.

[11] Guerrieri M, de Sanctis A, Crosta F (2007). Adrenal incidentaloma: Surgical update. *Journal of Endocrinological Investigation*.

[12] Strong VE, Kennedy T, Al-Ahmadie H (2008). Prognostic indicators of malignancy in adrenal pheochromocytomas: clinical, histopathologic, and cell cycle/apoptosis gene expression analysis. *Surgery*.

[13] Conzo G, Tricarico A, Belli G (2009). Adrenal incidentalomas in the laparoscopic era and the role of correct surgical indications: observations from 255 consecutive adrenalectomies in an Italian series. *Canadian Journal of Surgery*.

[14] Gagner M, Lacroix A, Bolte E (1992). Laparoscopic adrenalectomy in Cushing’s syndrome and pheochromocytoma. *The New England Journal of Medicine*.

[15] Miccoli P, Raffaelli M, Berti P, Materazzi G, Massi M, Bernini G (2002). Adrenal surgery before and after the introduction of laparoscopic adrenalectomy. *British Journal of Surgery*.

[16] Saunders BD, Wainess RM, Dimick JB, Upchurch GR, Doherty GM, Gauger PG (2004). Trends in utilization of adrenalectomy in the United States: have indications changed?. *World Journal of Surgery*.

[17] Lifante JC, Cenedese A, Fernandez Vila JM, Peix JL (2005). Impact of laparoscopy on the management of adrenal diseases. A retrospective study of 220 patients. *Annales de Chirurgie*.

[18] Sidhu S, Bambach C, Pillinger S (2002). Changing pattern of adrenalectomy at a tertiary referral centre 1970–2000. *ANZ Journal of Surgery*.

[19] Novitsky YW, Czerniach DR, Kercher KW, Perugini RA, Kelly JJ, Litwin DEM (2003). Feasibility of laparoscopic adrenalectomy for large adrenal masses. *Surgical Laparoscopy, Endoscopy and Percutaneous Techniques*.

[20] Dimas S, Roukounakis N, Kafetzis I (2007). Feasibility of laparoscopic adrenalectomy for large pheochromocytomas. *Journal of the Society of Laparoendoscopic Surgeons*.

[21] Henry J-F, Sebag F, Iacobone M, Mirallie E (2002). Results of laparoscopic adrenalectomy for large and potentially malignant tumors. *World Journal of Surgery*.

[22] Yoh T, Hosono M, Komeya Y (2008). Quantitative evaluation of norcholesterol scintigraphy, CT attenuation value, and chemical-shift MR imaging for characterizing adrenal adenomas. *Annals of Nuclear Medicine*.

[23] Israel GM, Korobkin M, Wang C, Hecht EN, Krinsky GA (2004). Comparison of unenhanced CT and chemical shift MRI in evaluating lipid-rich adrenal adenomas. *The American Journal of Roentgenology*.

[24] Jhaveri KS, Wong F, Ghai S, Haider MA (2006). Comparison of CT histogram analysis and chemical shift MRI in the characterization of indeterminate adrenal nodules. *The American Journal of Roentgenology*.

[25] Boland GWL, Blake MA, Hahn PF, Mayo-Smith WW (2008). Incidental adrenal lesions: principles, techniques, and algorithms for imaging characterization. *Radiology*.

